# Identification of a Novel Glycolysis-Related Gene Signature Correlates With the Prognosis and Therapeutic Responses in Patients With Clear Cell Renal Cell Carcinoma

**DOI:** 10.3389/fonc.2021.633950

**Published:** 2021-03-17

**Authors:** Zhengtong Lv, Lin Qi, Xiheng Hu, Miao Mo, Huichuan Jiang, Yuan Li

**Affiliations:** ^1^ Department of Urology, Beijing Hospital, National Center of Gerontology, Institute of Geriatric Medicine, Chinese Academy of Medical Sciences, Beijing, China; ^2^ Graduate School of Peking Union Medical College and Chinese Academy of Medical Sciences, Beijing, China; ^3^ Department of Urology, Xiangya Hospital, Central South University, Changsha, China

**Keywords:** clear cell renal cell carcinoma, TCGA, glycolysis, prognosis, gene set enrichment analysis (GSEA)

## Abstract

**Background:**

Accumulating evidences indicate significant alterations in the aerobic glycolysis in clear cell renal cell carcinoma (ccRCC). We aim to develop and validate a glycolysis-related genes signature for predicting the clinical outcomes of patients with ccRCC.

**Methods:**

mRNA expression profiling of ccRCC was obtained from The Cancer Genome Atlas database. Univariate Cox regression analysis and lasso Cox regression model were performed to identify and construct the prognostic gene signature. The protein expression levels of the core genes were obtained from the Human Protein Atlas database. We used four external independent data sets to verify the predictive power of the model for prognosis, tyrosine kinase inhibitor (TKI) therapy, and immunotherapy responses, respectively. Finally, we explored the potential mechanism of this signature through gene set enrichment analysis (GSEA).

**Results:**

Through the GSEA, glycolysis-related gene sets were significantly different between ccRCC tissues and normal tissues. Next, we identified and constructed a seven-mRNA signature (GALM, TGFA, RBCK1, CD44, HK3, KIF20A, and IDUA), which was significantly correlated with worse survival outcome and was an independent prognostic indicator for ccRCC patients. Furthermore, the expression levels of hub genes were validated based on the Human Protein Atlas databases. More importantly, the model can predict patients’ response to TKI therapy and immunotherapy. These findings were successfully validated in the external independent ccRCC cohorts. The mechanism exploration showed that the model may influence the prognosis by influencing tumor proliferation, base mismatch repair system and immune status of patients.

**Conclusions:**

Our study has built up a robust glycolysis-based molecular signature that predicts the prognosis and TKI therapy and immunotherapy responses of patients with ccRCC with high accuracy, which might provide important guidance for clinical assessment. Also, clinical investigations in large ccRCC cohorts are greatly needed to validate our findings.

## Introduction

Renal cell carcinoma (RCC) is one of the top ten cancers in the world, with about 65,000 new cases occurring each year in the United States (1). The most common and aggressive subtype is clear cell RCC (ccRCC), which accounts for about 80% of all RCC (2). ccRCC is usually asymptomatic in the early stages, with metastases occurring in about 25–30% of patients at the time of diagnosis (3). Because of the tumor heterogeneity, patients with the same degree of progression can show different prognosis and treatment responses (4). Therefore, it is necessary to find effective biomarkers to assess prognosis and identify potential patients at high risk for ccRCC.

One of the features of the cancer is metabolic reprogramming (5). Cancer cells have a high degree of glycolysis. It can convert glucose to lactic acid with or without oxygen, which called “Warburg effect” (6). Studies have shown that tumor glycolysis is a promising target for the treatment of cancer (7). Therefore, elucidating the relationship between glycolysis and tumor will help to better understand the mechanism of tumor formation and the development of ccRCC.

In this study, we used the database of The Cancer Genome Atlas (TCGA) to develop a seven-glycolysis-related genes signature to predict prognosis and therapeutic responses in ccRCC patients. The predictive power of the signature was successfully validated using four external ccRCC cohorts. These findings reveal the close relations between glycolysis and tumor prognosis and open up new ideas for the treatment of ccRCC.

## Materials and Methods

### Public Data Source

**Figure 1:**
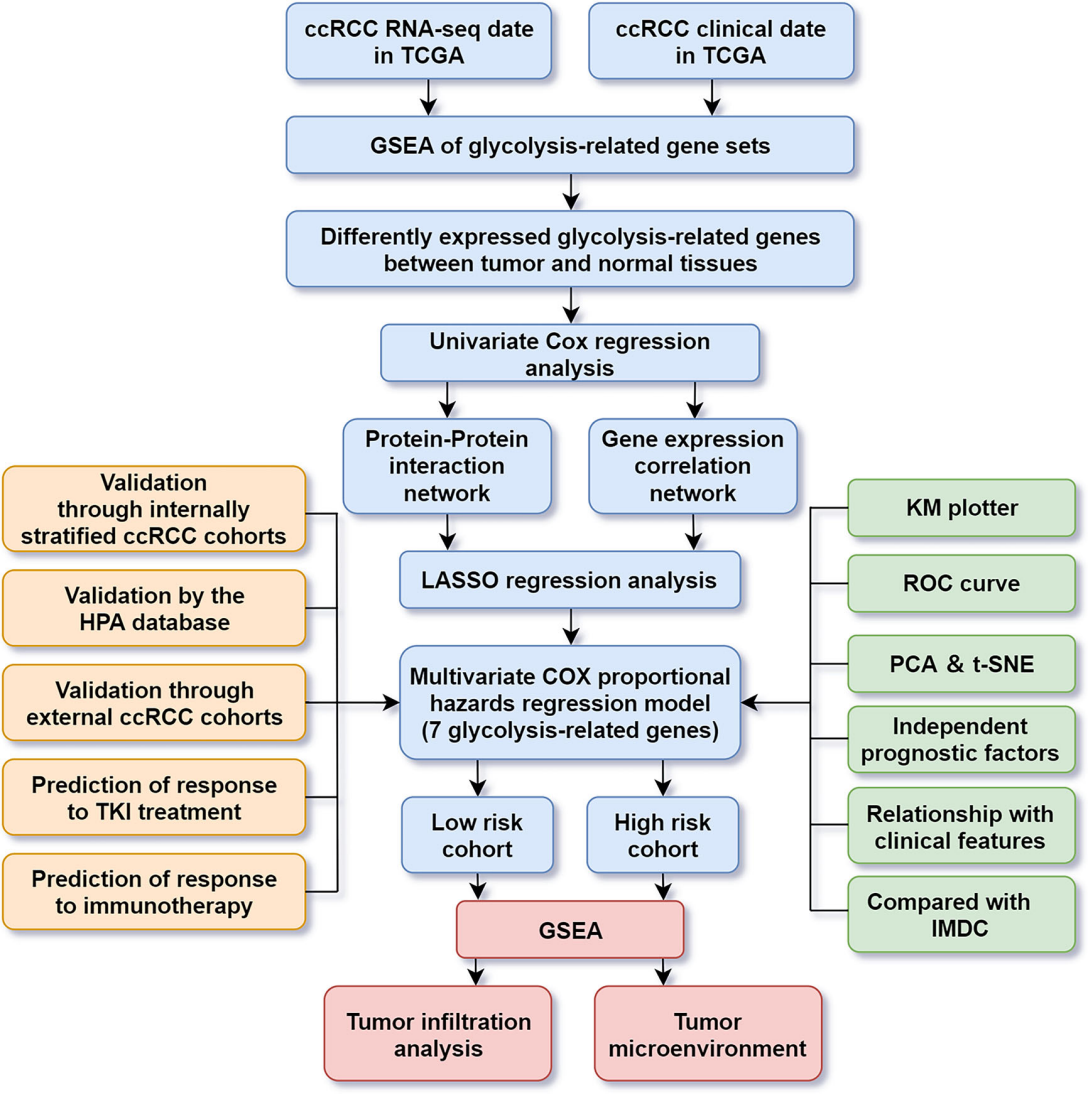
Flowchart for analyzing glycolysis-related gene in ccRCC.

The transcriptome and clinical data of ccRCC patients were acquired from project of TCGA (https://cancergenome.nih.gov/). Five hundred thirty-nine ccRCC cases and 72 normal control samples were included for subsequent analysis. The datasets of GSE22541 from the Gene Expression Omnibus were used to validate the model’s prediction of prognosis. The datasets of E-MTAB-3267 (8) and E-MTAB-3218 (9) from ArrayExpress were used to validate the model’s prediction of response to tyrosine kinase inhibitor (TKI) and immunotherapy, respectively. The Human Protein Atlas (HPA) database was used to observe the immunohistochemistry of genes with prognostic values (http://www.proteinatlas.org/). The flow chart of this study is shown in **Figure 1**.

### Gene Set Enrichment Analysis

We downloaded five glycolysis-related gene sets from the Molecular Signatures Database (“BIOCARTA GLYCOLYSIS PATHWAY”, “HALLMARK GLYCOLYSIS”, “KEGG GLYCOLYSIS GLUCONEOGENESIS”, “REACTOME GLYCOLYSIS”, and “REACTOME REGULATION OF GLYCOLYSIS BY FRUCTOSE 2 6 BISPHOSPHATE METABOLISM”). Gene set enrichment analysis (GSEA) was used to determine if these five gene sets were significantly different between the ccRCC and normal group. Total 1,000 times gene set permutations were performed to finally get the normalized enrichment score, normalized P-value and false discovery rate (FDR). Two hundred eighty-eight genes from these five glycolysis-related gene sets were identified as core genes. After constructing the prognosis model, we also used GSEA based on the gene sets of hallmarks and KEGG to identify enriched biological process significantly altered in high-risk cohort.

### Screening of Candidate Genes

Firstly, differentially expressed genes (DEGs) analysis was performed by “limma” package. Genes with a | log2 (FC) | >1 and FDR <0.05 were defined as DEGs. Then, we used univariate Cox regression analysis to identify overall survival (OS) associated genes. The genes, if P <0.05, were selected as candidate genes for subsequent construction of prognostic model.

### Establishing a Protein-Protein Interaction Network and Genes Expression Correlation Network

We used STRING (STRING: http://www.string-db.org/) to conduct the PPI network to illustrate the direct interaction of proteins among the proteins coding by the candidate genes. The network was presented by the Cytoscape software (https://cytoscape.org/). In addition, we also explored whether these candidate genes were correlated at the transcriptional level. The “igraph” and “reshape2” package in R software was used to conduct correlation network of candidate genes.

### Construction of Risk Prognostic Model

Then, Lasso regression was performed to eliminate the genes that were over-fitting with the model and further screen the potential hub genes. Finally, multivariate Cox regression was used to construct the prognostic risk models. We calculated each patient’s risk score according to the following formula: $Risk\text{ score =}\Sigma_{j=1}^{n}\;Coef_{j}\times Exp_{j}$, where Coef j and Exp j representing the coefficient and the gene relative expression. The median risk value was used as the cutoff value to divide 539 patients into high and low risk subgroups.

### The Validation Patient Cohort

Ethical approval was passed in Xiangya Hospital, Central South University about the validation cohort. The study specimens comprised of 78 patients with ccRCC. All of them signed informed consent forms. Tissues were collected within 10 min after surgical resection and rapidly protected in RNAlater and kept in liquid nitrogen for long-term storage. Additional clinical and transcriptome information is available in **Supplementary Materials**. Using TRIzol reagent (Vazyme, Nanjing, China), we got the total RNA from patients’ tissues, and then used SuperScript III Reverse Transcriptase (Invitrogen, Carlsbad, CA, USA) to reverse transcribed into cDNA. qRT-PCR was performed using SYBR-Green Premix (Vazyme) and specific PCR primers (Sangon Biotech Co., Ltd, Shanghai, China). GAPDH was selected for internal reference. The fold-changes value was got using 2^−ΔΔCt^ method. When the mRNA expression of the hub genes was obtained, the validated ccRCC cohort was divided into high- and low-risk groups based on the calculated risk score. Then the difference in OS was calculated, and we also evaluated the associations between risk score and the clinicopathologic factors of the validated ccRCC cohort. Finally, we also performed the univariate and multivariate Cox regression analyses to determine whether the risk score was an independent prognostic factor.

### Immune Infiltration and Tumor Microenvironment

Single sample Gene Set Enrichment Analysis (ssGSEA) was performed to quantify the immune infiltration levels using GSVA package (10). The annotated gene set file was obtained from the study of Jie-Ying Liang et al. (11). We finally quantified the enrichment levels of the 16 immune cells and 13 immune-related pathways in each ccRCC sample, and the results were expressed as immune scores. The boxplot would show the level of immune infiltration in the high and low risk group. Besides that, we used the ESTIMATE package to calculate immune/stromal/ESTIM scores to predict the tumor microenvironment (TME) in ccRCC (12). The Spearman rank test was used to analyze correlations between the risk score and immune/stromal/ESTIM scores.

### Statistical Analysis

All analyses were carried out by R software (version 3.6.1) and corresponding packages. Wilcoxon Rank-Sum test was used to detect the difference of gene expression between tumor and normal tissues. Kaplan-Meier curves and a log-rank test were used to check the significant difference in OS. The Receiver Operating Characteristic (ROC) analysis was used to examine the sensitivity and specificity of survival prediction. The area under the ROC curve (AUC) served as an index for prognostic efficacy. Univariate and multivariate analysis by COX regression show the independent prognostic factors. The difference significance was defined by P <0.05.

## Results

### Initial Screening of Candidate Gene Genes

**Figure 2:**
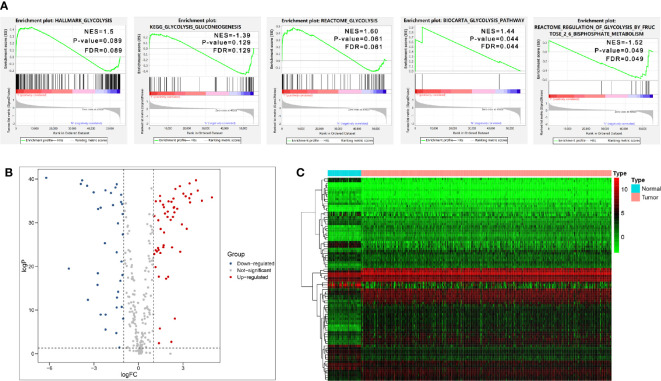
Initial screening of genes by GSEA and differential expression analysis. **(A)** GSEA results of the five glycolysis-related gene sets. **(B, C)** Volcano Plot and heat map of differential expression of glycolysis-related genes between ccRCC tissues and normal renal tissues.

**Figure 3:**
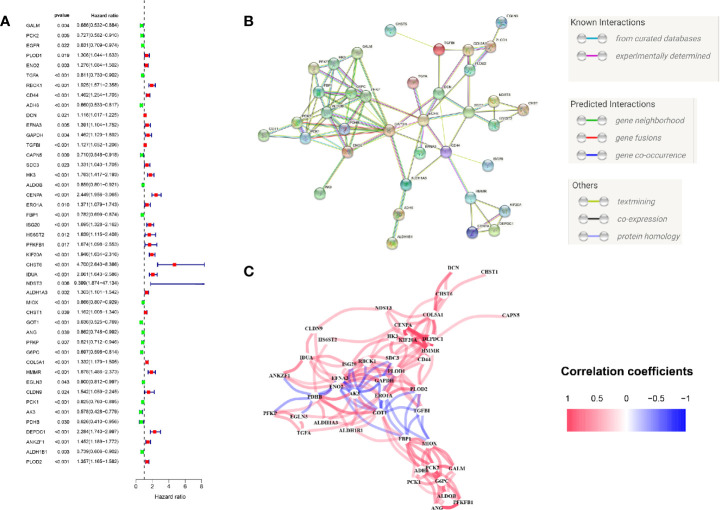
Initial screening of OS-related glycolysis-related genes. **(A)** 45 genes associated with OS of ccRCC patients were obtained through univariate Cox regression analysis. **(B)** The Construction of PPI network by STRING. **(C)** The genes expression correlation network.

We firstly performed GSEA to explore whether five glycolysis-related gene sets were significantly different between ccRCC and normal samples. The results showed that all these five gene sets were significantly enriched in ccRCC samples (FDR <0.25), especially in BIOCARTA GLYCOLYSIS PATHWAY gene set and REACTOME REGULATION OF GLYCOLYSIS BY FRUCTOSE 2 6 BISPHOSPHATE METABOLISM gene set (P < 0.05) (**Figure 2A**). Then we collected 288 participating genes on the Glycolysis pathway and finally found total 90 DEGs, in which 37 were downregulated and 53 were upregulated in ccRCC tissues compared with normal renal tissues (**Figures 2B, C**). Next, univariate Cox regression analysis was performed for preliminary screening and obtained 45 genes associated with OS from the 90 DEGs (p < 0.05) (**Figure 3A**). We uploaded these 90 OS-related genes s to STRING to construct a PPI network (**Figure 3B**). In terms of the mRNA expression levels of these genes, correlation analysis showed that these genes were strongly correlated at the transcriptional level (**Figure 3C**).

### Construction of the Seven-mRNA Signature to Predict Patient Outcomes

**Figure 4:**
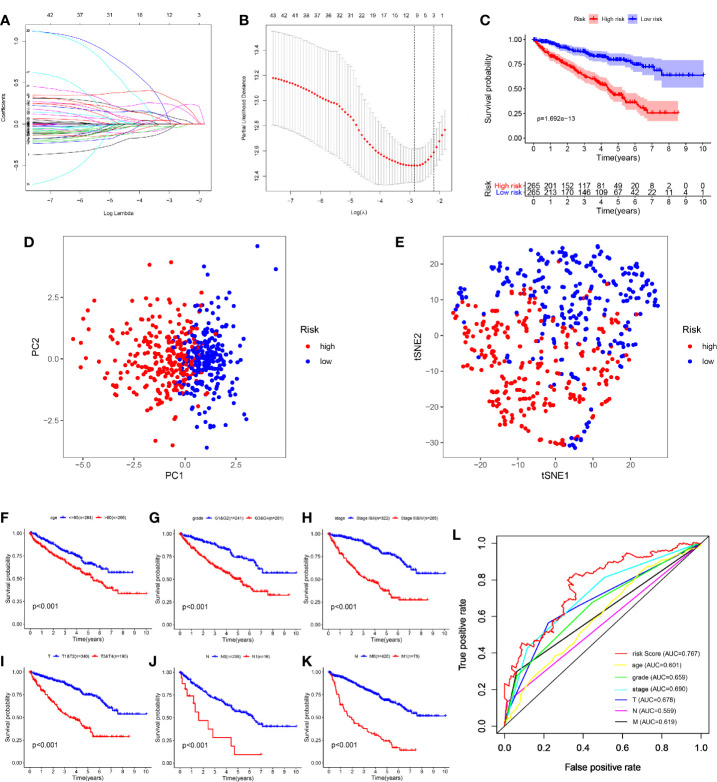
Construction and evaluation of the seven-mRNA signature. **(A, B)** A seven-mRNA signature was constructed by LASSO Cox regression. **(C)** The survival analysis of the two subgroups stratified based on the median of risk scores calculated by the risk model. **(D, E)** PCA and t-SNE analysis of the TCGA cohort. **(F–K)** Kaplan–Meier analysis of subgroup patients based on some clinicopathological features including age, grade, stage and TNM stage. **(L)** ROC curve of model and clinical characteristics predicting 5-year survival based on TCGA training set.

As these 45 OS-related genes may be collinear rather than independently, we performed the LASSO Cox regression to determine the real OS-affecting factors and finally identified a prognostic panel of seven glycolysis-related genes. The calculation formula of risk score is: Risk score = GALM * (-0.364) + TGFA * (-0.134) + RBCK1 * (0.194) + CD44 * (0.139) + HK3 * (0.2) + KIF20A * (0.359) + IDUA * (0.428). Among them, RBCK1, CD44, HK3, KIF20A and IDUA were risk factors and GALM, TGFA were protective factors (**Figures 4A, B**). We calculated each patient’s risk score and divided them into high- and low-risk group based on the median of risk score. Patients in the high-risk group had a higher risk of death (**Figure 4C**).

According to the expression of these seven hub genes, we performed dimensionality reduction in all patients and presented them with the methods of principal component analysis (PCA) and t-distributed stochastic neighbor embedding (t-SNE). Both PCA and t-SNE analysis suggest that different risk subgroups show significant discrete tendency directly in the two-dimensional plane (**Figures 4D, E**).

Traditional clinical indicators, such as age, grade, stage and TNM stage, can also distinguish high and low-risk patients (**Figures 4F–K**). When evaluating survival prediction, we found the 5-year AUC of our signature was 0.767, which was obviously higher than age (AUC = 0.601), grade (AUC = 0.659), stage (AUC = 0.690), T stage (AUC = 0.678), N stage (AUC = 0.559), and M stage (AUC = 0.619) (**Figure 4L**).

**Figure 5:**
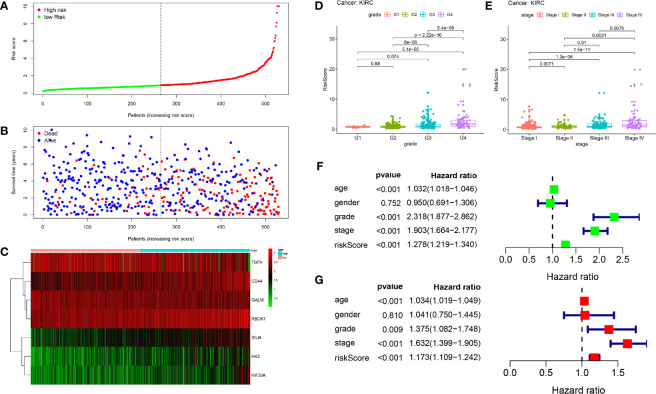
Prognostic analysis of seven-gene signature in the training set. The dotted line represented the median risk score and divided the patients into low- and high-risk group. **(A)** The curve of risk score. **(B)** Survival status of the patients. More dead patients corresponding to the higher risk score. **(C)** Heatmap of the expression profiles of the seven prognostic genes in low- and high-risk group. **(D, E)** The higher the risk score, the higher the tumor grade and stage. **(F, G)** Univariate and multivariate Cox regression analysis identified the indicators that significantly correlated with OS and revealed the independent prognostic factors.

The patients were arranged in ascending order according to the risk score (**Figure 5A**). **Figure 5B** showed the survival time of each patient. High-risk patients had a higher mortality than those in the low-risk group. Additionally, a heatmap displayed the expression profiles of nine mRNAs (**Figure 5C**). We further explored the relationship between the risk signature and other clinical features including age, grade, stage and TNM stage. We noticed that the higher the risk score, the higher the tumor grade and stage (**Figures 5D, E**). Not only that, we found that clinical indicators were significantly related with the hub genes of signature except GALM (**Table 1** and **Supplemental Materials**).

**Table 1:** The relationship between the risk signature and clinical variables.

Genes	Age	Gender	Grade	Stage	T	N	M
	<=65/>65	Female/Male	G1&G2/G3&G4	Stage I&II/III& IV	T1&T2/T3&T4	N0/ N1	M0/ M1
	T (P)	T (P)	T (P)	T (P)	T (P)	T (P)	T (P)
GALM	0.771 (0.441)	1.905 (0.058)	-0.416 (0.677)	-0.483 (0.630)	-0.105 (0.916)	-1.063 (0.302)	-0.035 (0.973)
TGFA	3.486 (5.329e-04)*	-0.812 (0.417)	3.485 (5.334e-04)*	2.724 (0.007)*	2.197 (0.029)*	0.674 (0.510)	3.215 (0.002)*
RBCK1	-8.052 (6.516e-15)*	-2.472 (0.014)*	-8.107 (3.968e-15)*	-6.924 (1.863e-11)*	-6.671 (1.054e-10)*	-2.263 (0.038)*	-4.247 (4.861e-05)*
CD44	-3.334 (9.197e-04)*	-2.743 (0.006)*	-3.636 (3.041e-04)*	-3.143 (0.002)*	-3.474 (5.83e-04)*	-2.67 (0.018)*	-2.676 (0.009)*
HK3	-3.73 (2.131e-04)*	0.897 (0.370)	-4.435 (1.125e-05)*	-4.293 (2.262e-05)*	-4.186 (3.683e-05)*	-2.665 (0.018)*	-3.547 (5.958e-04)*
KIF20A	-6.011 (3.719e-09)*	-3.102 (0.002)*	-6.895 (1.688e-11)*	-6.871 (3.314e-11)*	-6.529 (3.085e-10)*	-4.087 (9.798e-04)*	-5.532 (2.747e-07)*
IDUA	-3.597 (3.523e-04)*	2.021 (0.044)*	-3.965 (8.362e-05)*	-3.843 (1.423e-04)*	-3.241 (0.001)*	-2.127 (0.050)	-3.28 (0.001)*
riskScore	-5.912 (8.969e-09)*	-0.637 (0.525)	-6.421 (4.866e-10)*	-5.189 (4.54e-07)*	-4.845 (2.443e-06)*	-3.059 (0.008)*	-3.576 (5.883e-04)*

*P < 0.05.

Univariate and multivariate Cox regression analyses were conducted to assess whether the model was an independent predictor among other clinical factors including age, gender, grade and stage. We found the risk score remained independently associated with OS not only at univariate but also multivariate analysis when combined with all the clinical features (P < 0.05) (**Figures 5F, G**).

### Validation of the Prognostic Signature Through Internally Stratified Clear Cell Renal Cell Carcinoma Cohorts

**Figure 6:**
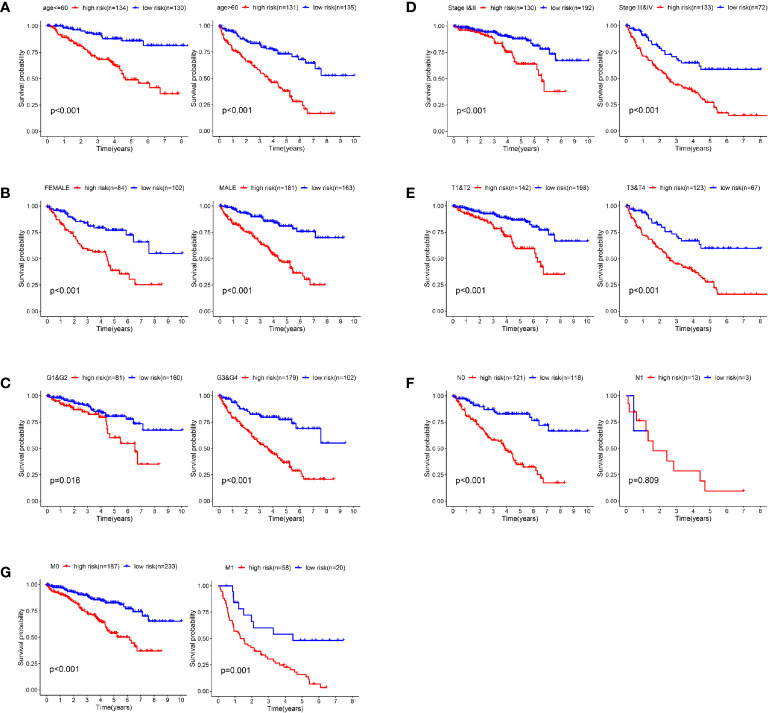
Validation of the prognostic signature through internally stratified ccRCC cohorts. **(A–G)** Kaplan–Meier curves showed that the high-risk group had shorter OS than the low-risk group in almost all subgroups.

In order to verify the predictive efficiency in stratified cohorts, we stratified patients with ccRCC into two subgroups according to age (≤ 60 or >60 years), gender (female or male), grade (G1&G2 or G3&4), stage (Stage I&II or Stage III&IV), T stage (T1&T2 or T3&T4), N stage (N0 or N1) and M stage (M0 & M1). Kaplan–Meier curves showed that the high-risk group had shorter OS than the low-risk group in all subgroups except N1 stage subgroup. There were only 16 patients in the N1 subgroup, so differences in survival could not be obtained (**Figures 6A–G**).

### Validation of the Prognostic Signature Through HPA Database and External Clear Cell Renal Cell Carcinoma Cohorts

**Figure 7:**
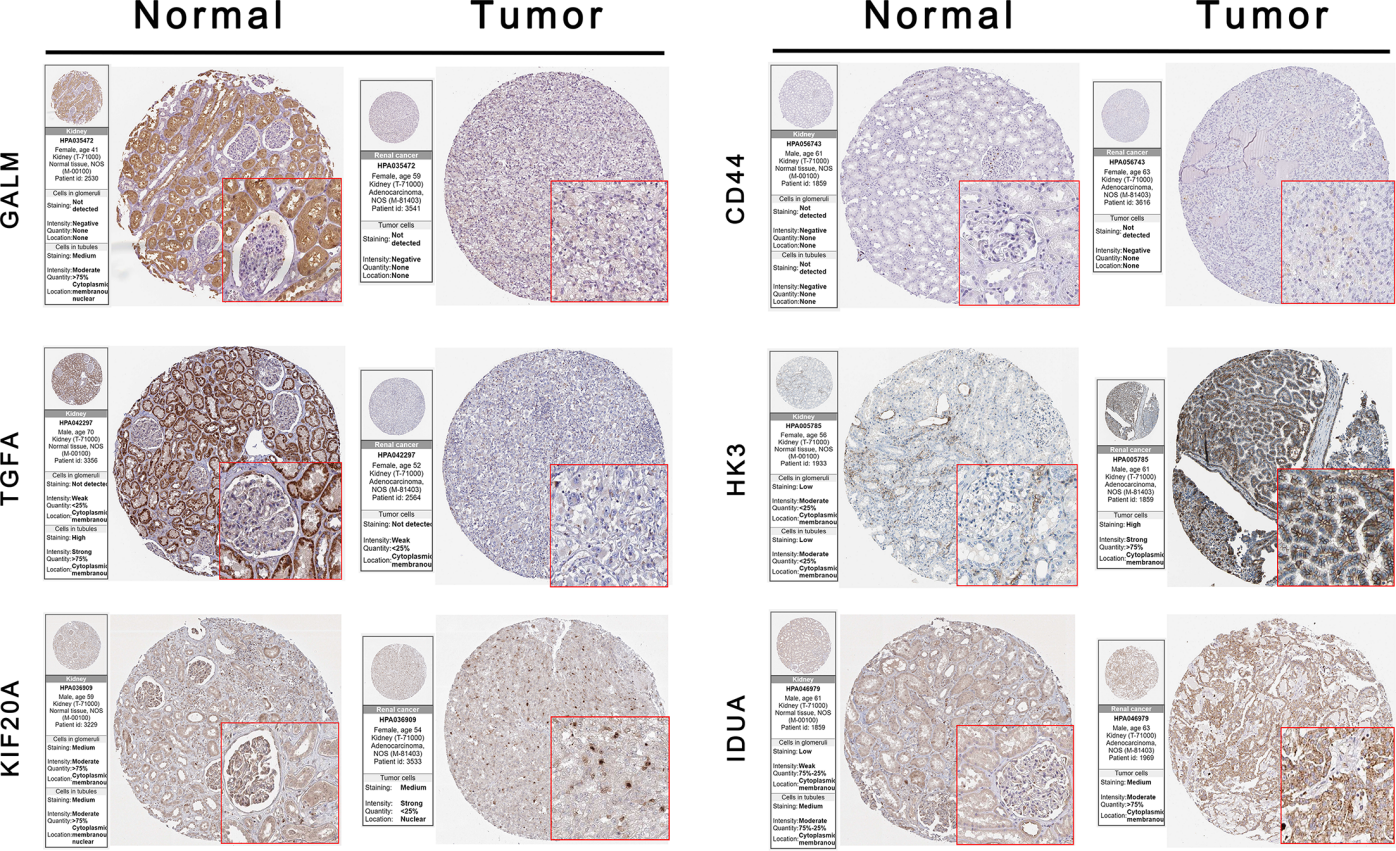
Validation the expression of the signature genes between ccRCC and normal tissues on translational level by the HPA database. When compare tumor samples to paired adjacent normal samples, the expression difference of these seven core genes were consistent in external ccRCC cohort.

We used the HPA database to confirm the protein expression between ccRCC and normal tissues. The protein expression of CD44, HK3, KIF20A, and IDUA was higher in the tumor tissues compared to the normal tissue, and the protein expression of GALM and TGFA was lower in tumor tissues than normal, which was consistent with our results in TCGA. However, RBCK1 was not found in the database (**Figure 7**).

**Figure 8:**
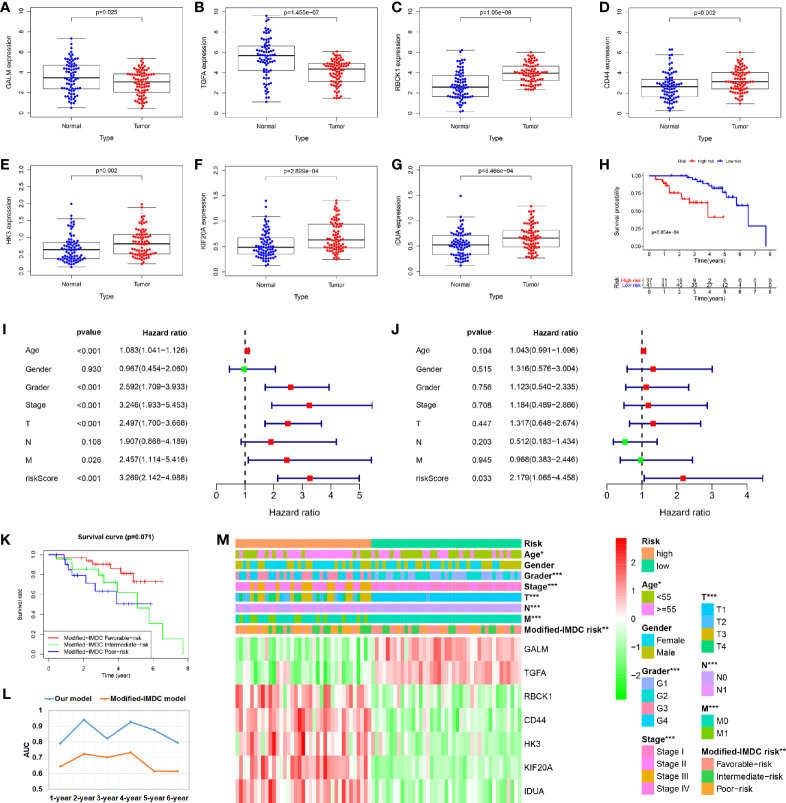
Validation of the prognostic signature through external ccRCC cohort. **(A–G)** When compare tumor samples to paired adjacent normal samples, the expression difference of these seven core genes were consistent with the TCGA cohort results. **(H)** Kaplan–Meier curves also showed that the OS was significantly shorter in the high-risk group compared to that in the low-risk group. **(I, J)** Univariate and multivariate Cox regression analyses showed that the risk score had prominent prognostic values. **(K)** Kaplan-Meier curves for OS by modified-IMDC subgroups. **(L)** Comparison of AUC values for predicting long-term survival between the two prognostic models. **(M)** Correlations between risk model and clinicopathological features.

A total of 78 cases were included in the external validated ccRCC cohort. We found that the expression levels of these seven core genes were significantly different between ccRCC samples and paired adjacent normal samples by qRT-PCR (P < 0.05) (**Figures 8A–G**). Based on the cut-off value of the risk scores, all patients were categorized into high-risk group and low-risk group. Survival analysis showed that OS in high-risk group was significantly shorter than that in low-risk group (P < 0.05) (**Figure 8H**). Univariate and multivariate Cox regression analyses showed that the risk score had prominent prognostic values (**Figures 8I, J**). The International Metastatic RCC Database Consortium (IMDC) model is the most widely used risk assessment tool in metastatic RCC assessment, assisting in treatment decision-making and predicting prognosis (13). Hannah et al. constructed a modified-IMDC risk classification system, which can be applied for patient with non-metastatic ccRCC (14). Through the modified-IMDC risk classification system, 33 patients were classified as favorable-risk group, 23 as intermediate-risk group, and 22 as poor-risk group. The prognosis of the three groups was different, although P value was not significant. (**Figure 8K**). In order to evaluate the predictive power of our model compared to modified-IMDC, we calculated the AUC values for different survival time predictions. The AUC value of our model was more accurate than modified-IMDC in predicting any survival time (**Figure 8L**). Moreover, our risk grouping system was significantly correlated with tumor grade and stage, TNM staging, and modified-IMDC risk classification system (**Figure 8M**).

**Figure 9:**
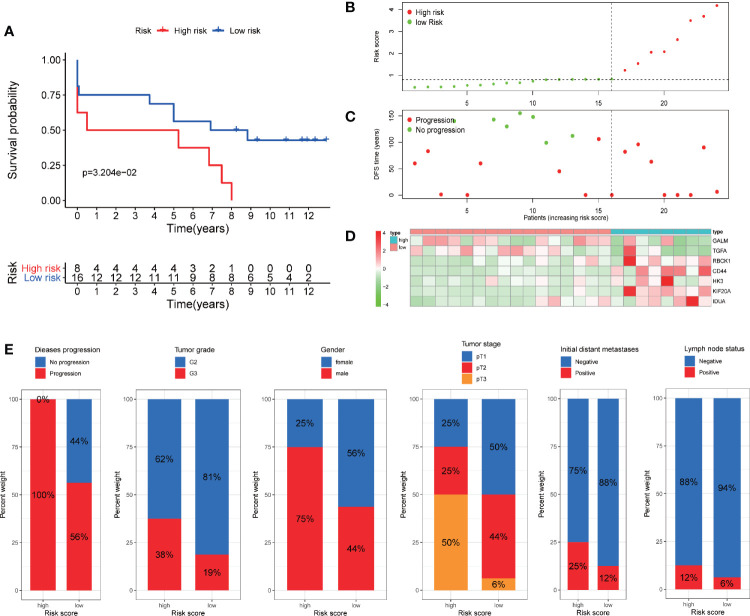
Validation of the prognostic signature through GSE22541 cohort. **(A)** Kaplan–Meier curves also showed that the DFS was significantly shorter in the high-risk group compared to that in the low-risk group. **(B)** The curve of risk score. **(C)** Disease progression status of the patients. More progressive patients corresponding to the higher risk score. **(C)** Heatmap of the expression profiles of the seven prognostic genes in low- and high-risk group.

In addition, we verify the predictive ability of the model in another external independent dataset (GSE22541). GSE22541 contains complete transcriptome and clinical information of 24 patients with ccRCC. Based on the cut-off value of the risk scores in TCGA cohort, all patients were also categorized into high-risk group and low-risk group (**Figure 9B**). Survival analysis showed that disease-free survival (DFS) time in high-risk group was significantly shorter than that in low-risk group (P <0.05) (**Figure 9A**). All patients in the high-risk group had disease progression, while all patients without disease progression were classified into the low-risk group (**Figure 9C**). It was also observed that GALM and TGFA were highly expressed as protective factors in the low-risk group, while RBCK1, CD44, HK3, KIF20A, and IDUA were highly expressed as risk factors in the high-risk group (**Figure 9D**). At the same time, we observed that many clinicopathological features were significantly different between the high and low risk groups. The high-risk group had more disease progression, higher grade tumor, more male patients, and higher TNM stage than the low-risk group (**Figure 9E**).

### Prediction of Tyrosine Kinase Inhibitor Therapy and Immunotherapy Response

**Figure 10:**
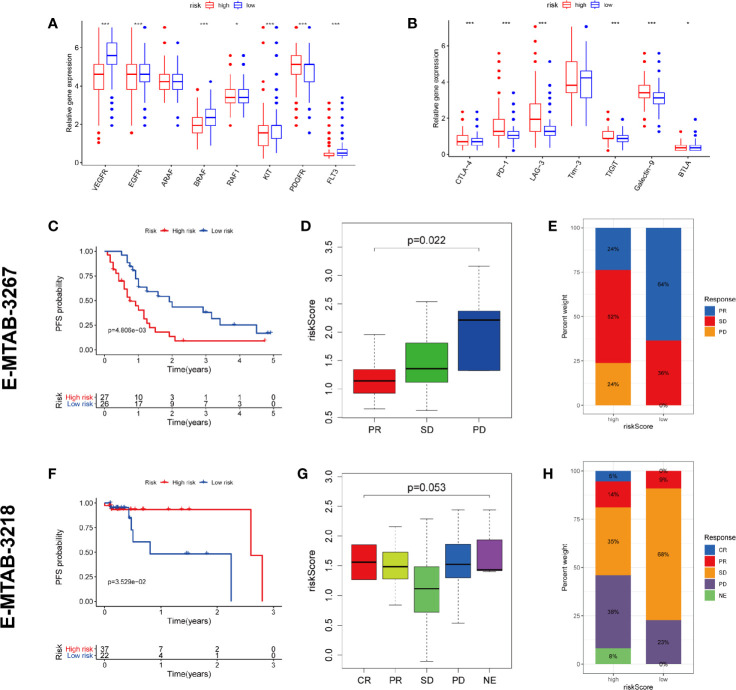
Prediction of TKI therapy and immunotherapy response. **(A)** The difference of TKI target genes expression between high and low risk group. **(B)** The difference of immune checkpoint genes expression between high and low risk group. **(C–E)** The predictive power of risk scores in response to TKI therapy. **(F–H)** The predictive power of risk scores in response to immunotherapy. CR, complete response; PR, partial response; SD, stable disease; PD, progressive disease; NE, Not Evaluated. (***p < 0.001; **p < 0.01; *p < 0.05).

TKI therapy and immunotherapy are important treatments for metastatic RCC and are strongly recommended by guidelines. We tried to explore whether our prognostic model has potential predictive value for response to both therapies. Firstly, we detected the expression of targeted drug-related genes and immune checkpoint genes in high and low risk patients. We found that the expression of many TKI-related genes (VEGFR, EGFR, BRAF, RAF1, KIT, and FLT3) in high-risk patients was significantly lower than that in the low-risk group (except PDGFR), potentially suggesting that high-risk patients may have a poor response to TKI therapy (**Figure 10A**). On the contrary, almost all of the immune checkpoint genes (CTLA-4, PD-1, LAG-3, TIGIT, Galectin-9, and BTLA) were expressed higher in the high-risk group than in the low-risk group, which potentially suggests that the high-risk group may have a better response to immunotherapy (**Figure 10B**).

To test our conjecture, we chose two external independent data sets E-MTAB-3267 and E-MTAB-3218. The former included 53 patients with metastatic ccRCC treated with sunitinib, while the latter included 59 patients with metastatic ccRCC treated with nivolumab. Both studies have detailed transcriptome information, drug response information, and prognosis information. The verification results are highly consistent with our conjecture. The high-risk population predicted by our model has worse PFS than the low-risk population in receiving TKI therapy (**Figure 10C**). The lower the risk score, the higher the likelihood of a response to TKI treatment (**Figure 10D**). Compared with the high-risk group, the low-risk group has a higher proportion of treatment response rate (64 vs. 24%) (**Figure 10E**). On the contrary, the high-risk population predicted by our model has better PFS than the low-risk population in receiving immunotherapy (**Figure 10F**). The higher the risk score, the higher the likelihood of a response to immunotherapy (**Figure 10G**). Compared with the low-risk group, the high-risk group has a higher proportion of treatment response rate (19 vs. 9%) (**Figure 10H**). These validation results fully demonstrate that our predictive model also has the ability to predict TKI therapy and immunotherapy response.

### Further Analysis of High- and Low-Risk Cohorts

**Figure 11:**
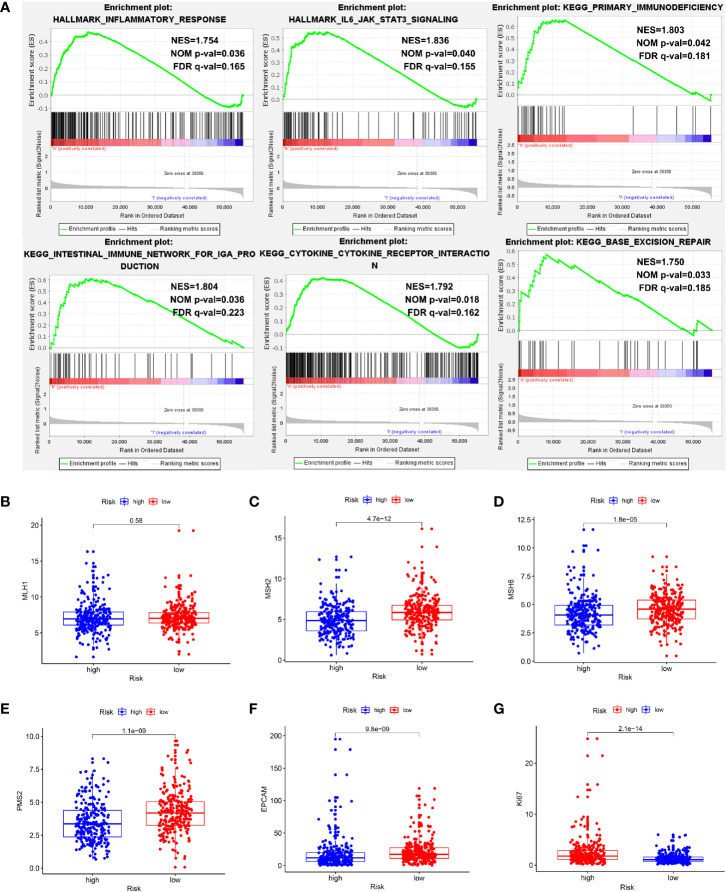
Exploration of potential mechanisms of this signature. **(A)** The GSEA showed that the base mismatch repair, immune related and inflammation related pathways were significant enriched in high-risk patients. **(B–F)** Four of five MMRs genes (MSH2, MSH6, PMS2, and EPCAM) were low expressed in the high-risk cohort (P < 0.05). **(G)** The Ki67 expression level was higher in high-risk patients (P < 0.05).

To explore the mechanisms underlying our risk model, we subsequently conducted biological process and pathway analysis using GSEA. GSEA analysis revealed significant enrichments in the base mismatch repair, immune related and inflammation related pathways in high-risk cohort (**Figure 11A**) (P < 0.05, FDR < 0.25). The mismatch repair system (MMRs) is an intracellular mismatch repair mechanism, in which the loss of key gene function leads to DNA replication errors leading to the production of higher somatic mutations, which may lead to the development of tumors. We compared the expression differences of five MMRs genes (MLH1, MSH2, MSH6, PMS2, and EPCAM) in high and low risk populations, and found that four MMRs genes (MSH2, MSH6, PMS2, and EPCAM) were low expressed in the high-risk cohort, suggesting that the mismatch repair mechanism in high-risk populations may be inhibited by tumors (**Figures 11B–F**). In addition, we also compared the Ki67 expression levels in high- and low-risk patients, and found that the Ki67 expression level was higher in high-risk patients (**Figure 11G**), indicating the more active tumor cell proliferation, the faster tumor growth, and the poorer tissue differentiation in high-risk patients.

### Immune Infiltration and Tumor Microenvironment

**Figure 12:**
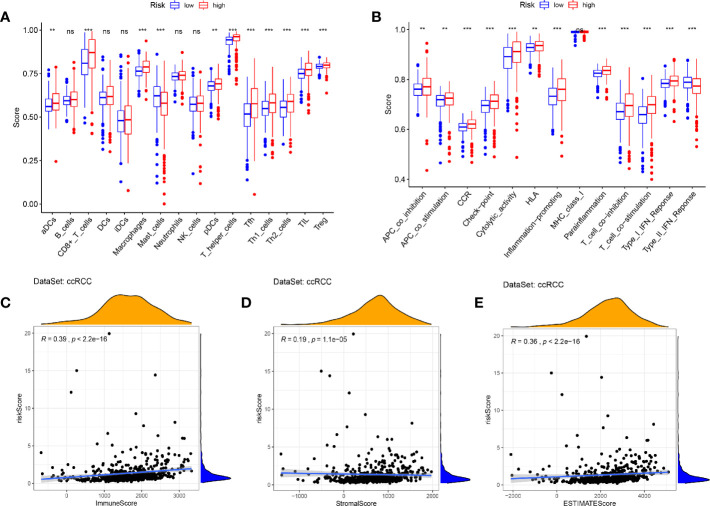
Immune infiltration and tumor microenvironment in the high- and low- risk cohorts. **(A, B)** The ssGSEA results showed that the high-risk cohort had higher levels of immune cells infiltration and more active immune-related functions than the low-risk cohort (P < 0.05). **(C–E)** With the increase of risk score, the immune/stromal/ESTIM scores have also increased by the “ESTIMATE” package. (***p < 0.001; **p < 0.01; *p < 0.05).

In view of the GSEA results showing that immune and inflammatory processes were significantly enriched in the high-risk cohort, we compared the differences in immune infiltration between the high and low risk cohorts by the method of ssGSEA. The results showed that the high-risk cohort had higher levels of immune cells infiltration (except for B cells, DCs, iDCs, Maste cells, Neutrophils, and NK cells) and more active immune-related functions (except for MHC class I and Type II IFN Response) than the low-risk cohort (**Figures 12A, B**). We used the ESTIMATE package to assess the TME and came to similar results. With the increase of risk score, the immune/stromal/ESTIM scores as prediction of TME have also increased (**Figures 12C–E**).

## Discussion

As the first step in the catabolism of most carbohydrates, glycolysis of cellular respiration is a complex reaction. Most glycolysis occurs in the cytoplasm, which is characterized by the absence of any oxygen molecules. As the main energy source of cancer cells, increased glycolysis can produce ATP for cancer cells, which contributes to the growth and metabolism of cancer cells (15, 16). More and more evidence showed that tumor glycolysis also played a key role in stimulating immunosuppressive networks, which are crucial for cancer cells to escape immune surveillance (17). So, the application of small molecules to inhibit key enzymes in the glycolytic pathway provides a new field for cancer research (18). Therefore, glycolysis may be an emerging marker and a potential prognostic indicator of malignant tumors.

It is really the fact that several glycolysis-related mRNAs have been identified as biomarkers of tumor prognosis. Lei Zhang et al. developed a nine-gene glycolysis-related risk signature that can predict prognosis in lung adenocarcinoma patients (19). Chen Zhang et al. constructed a four-mRNA glycolysis-based signature with bladder cancer (20). Zihao Wang et al. identified a nine-gene risk profile associated with glycolysis which predicts the prognosis of endometrial cancer (21). Longyang Jiang et al. also developed a glycolysis-related gene signature in hepatocellular carcinoma patients (22). So far, no studies have attempted to construct a glycolysis-related prognostic model of ccRCC. Morphologically, ccRCC cells are rich in lipids and glycogen, suggesting changes in fatty acid and glucose metabolism during ccRCC development (23). Indeed, more significant glycolysis dependence is observed in ccRCC (24), which provides an opportunity worth exploring for the development of new and more effective prognostic model (25).

The rapid development of high-throughput gene sequencing technology makes large-scale biodata research possible. In this study, we aimed to explore a new genetic marker to predict the prognosis of ccRCC. Firstly, we conducted GSEA and identified that glycolytic gene sets were significantly enriched in ccRCC tissues compared with paired normal tissues, which laid a theoretical foundation for the subsequent model construction. Subsequently, we identified a combination of seven genes instead of a single gene with prognostic value for ccRCC by univariate Cox-Lasso-multivariate Cox regression analyses. Furthermore, through comparison with some clinicopathological features such as age, histological grade and pathological stage, we found that our identified risk signature can strongly predict the prognosis. Univariate and multivariate Cox regression analyses demonstrated that the risk score calculated by the signature was an independent risk factor for ccRCC prognosis. We also analyzed the relationship between genes in the model and certain clinical variables (age, sex, histological grade, and pathological stage). We found that most genes in the model correlated positively with the progression of ccRCC. Consistent results of these hub genes at the protein expression level were also obtained by the HPA database. Not only that, we found that this model has a strong predictive efficacy through the validation of internally stratified ccRCC cohorts and two external ccRCC cohort.

TKI targeting the VEGF/VEGFR axis and immunotherapy targeting PD-1/PD-L1 have become the referral standard treatment of metastatic ccRCC. These combinations are now recommended in first line setting for metastatic ccRCC, according to the last European recommendations (26). Despite the encouraging activity and tolerable toxicity of the two therapies, the clinical benefits of individual patients are highly unpredictable, and sustained complete remission still exists in minority of cases. Therefore, many studies were devoted to finding biomarkers that can predict the response of these two therapies. Several studies have identified promising predictive biomarkers for TKI therapy, including tumor-infiltrating neutrophils (27), tumor-infiltrating CD19(+) B lymphocytes (28), circulating CD45(dim) CD34(+) VEGFR2(+) progenitor cells (29), expression of HLA class I (30), Carbonic anhydrase 9 (31), Serum amyloid alpha (32), genetic polymorphism in CTLA-4 (33) and so on. Similarly, immunotherapy also has some response biomarkers, including Polybromo 1 mutation, PD-L1 expression, tumor microenvironment, circulating T cells, neutrophil to lymphocytes ratio, IMDC classification and so on (34, 35). However, so far, it seems that no biomarker can accurately identify the efficacy of immunotherapy and/or TKI therapy, as many patients with negative biomarkers may respond to these treatments. Some critics think that a single biomarker is not enough to guide the choice of treatment, we need a comprehensive combination of biomarkers. Therefore, mRNA panel signatures or molecular subsets, reflecting the tumor as well as its microenvironment and the host, was given particular attention (35, 36).

In this study, we found that there were significant differences in TKI related genes and immune checkpoint genes between high-risk and low-risk groups, suggesting that the model has the potential to predict TKI and immunotherapy response. This conjecture was confirmed by two external datasets of metastatic ccRCC patients receiving sunitinib and nivolumab therapy respectively. We found that high-risk patients had a poor response to TKI therapy, but a better response to immunotherapy. So, the risk model may help to guide appropriate treatment in metastatic RCC patients.

In order to clarify the potential rational mechanism of this prognostic model, we performed GSEA analysis to identify the enriched biological process and pathway in high-risk cohort compared with low-risk cohort. The GSEA showed that the base mismatch repair, immune related and inflammation related pathways were significantly enriched in high-risk cohort. In view of this, we analyzed the differences in Ki67, MMRs genes, immune infiltration and tumor microenvironment between the high and low risk groups. We found that the Ki67 expression was higher, MMRs genes expression was lower in high-risk patients. This illustrated more active tumor cell proliferation (37) and inhibition of DNA repair mechanisms (38, 39) exist in high-risk patients. Metabolic recombination and immune evasion, two of the hallmarks of cancer, are distinct processes, but new research suggests a strong link (17, 40, 41). Metabolic competition between tumor and immune cells may lead to tumor immunosuppression (40). We did confirm this, the higher the patient’s risk score, the higher infiltration of immune cells, the more active immune-related functions and higher immune/stromal/ESTIM scores. The highly glycolytic tumors presented an immune-stimulatory tumor microenvironment, which has been proposed to predict immunotherapy response (42). This further supports our findings that high-risk patients respond better to immunotherapy despite poor prognosis.

By reviewing the existing studies, we found that these seven genes are indeed closely related to cancer in the field of basic or clinical medical research. For example, X Liu et al. found that knockdown of TGFA led to the suppression of proliferation in non-small cell lung cancer cell (43). Junfeng Zhang et al. found the formation of gastric cancer was related to TGFA gene polymorphisms (44). RBCK1 contributes to chemoresistance and stemness in colorectal cancer. RBCK1 can regulate ERalpha-positive breast cancer cell cycle progression and proliferation by supporting transcription of ERalpha and cyclin B1 (45), and could be a predictive marker of response to endocrine therapy in breast cancer (46). The role of CD44 and KIF20A in tumors has been well studied, and they are upregulated in a variety of cancers. The function of CD44 can induce EMT, alter cytoskeleton, and promote drug resistance and anti-apoptosis (47). A meta-analysis of 25 studies showed that patients with high KIF20A expression tended to have shorter OS than patients with low KIF20A expression (HR = 1.77, 95%CI = 1.57–1.99, P < 0.001) (48). Elena A Pudova et al. found that the overexpression of HK3 was associated with EMT in colorectal cancer (49). HK3 is also correlated with immune infiltrates and can predict immunotherapy response in non-small cell lung cancer (50). GALM and IDUA have not been thoroughly studied in tumors and it is worth studying in the future.

Inevitably, our study also had some shortcomings. This study cannot avoid the selection bias caused by retrospective characteristics. The sample size of validation data set is too small. Therefore, the prediction model needs to be further verified in large prospective clinical trials. The mechanism of glycolysis related genes affecting the occurrence and development of ccRCC needs further study *in vivo* and *in vitro*.

## Conclusion

We developed a seven-gene risk profile (GALM, TGFA, RBCK1, CD44, HK3, KIF20A, and IDUA) associated with glycolysis to predict the prognosis of ccRCC patients. The higher the risk parameters, the worse the prognosis. The higher the risk score, the worse the response to TKI therapy, but the better the response to immunotherapy. This signature can be used as a novel tool for predicting the clinical outcome of ccRCC, but also help to understand the mechanism of cell cellular glycolysis in carcinogenesis. Of course, the model still needs to be validated in prospective clinical trials with large sample sizes in the future.

## Data Availability Statement

The original contributions presented in the study are included in the article/**Supplementary Material**. Further inquiries can be directed to the corresponding author.

## Ethics Statement 

The studies involving human participants were reviewed and approved by The Institutional Ethics Committee of Xiangya Hospital, Central South University. The patients/participants provided their written informed consent to participate in this study.

## Author Contributions

ZL developed the methodology, made the formal analysis, and wrote–the original draft. LQ acquired the funding and supervised the study. XH acquired the funding and supervised the study. MM provided the software. HJ was in charge of the data curation. YL acquired the funding, wrote, reviewed, and edited the article, and conducted the project administration. All authors contributed to the article and approved the submitted version.

## Funding

This work is supported by the following grants: The National Natural Science Foundation of China (No. 81874094, No. 81974397, and No. 81902605), Hunan Provincial Natural Science Foundation of China (No. 2019JJ40484), and Science and Technology Plan Projects of Changsha City (kq1801114).

## Conflict of Interest

The authors declare that the research was conducted in the absence of any commercial or financial relationships that could be construed as a potential conflict of interest.

## Abbreviations

ccRCC, Clear cell renal cell carcinoma; RCC, Renal cell carcinoma; GSEA, Gene set enrichment analysis; TCGA, The Cancer Genome Atlas; FDR, False discovery rate; TCGA, The Cancer Genome Atlas; FDR, False discovery rate; DEGs, Differentially expressed genes; OS, Overall survival; PPI, Protein-protein interaction; PCA, Principal component analysis; t-SNE, t-distributed stochastic neighbor embedding; ROC, Receiver operating characteristic; AUC, The area under the ROC curve; HPA, Human Protein Atlas database; DFS, Disease-free survival; MMRs, Mismatch repair system; TKI, Tyrosine kinase inhibitor; TME, Tumor microenvironment; ssGSEA, Single sample Gene Set Enrichment Analysis.
