# Supplementary material for: Identification of a Novel Glycolysis-Related Gene Signature Correlates With the Prognosis and Therapeutic Responses in Patients With Clear Cell Renal Cell Carcinoma
Source: Front Oncol. 2021 Mar 17;11:633950. doi: 10.3389/fonc.2021.633950 (PMC8010189; doi:10.3389/fonc.2021.633950)
Supplement: Supplementary file 2 [file DataSheet_2.docx]

**Supplementary figures:**


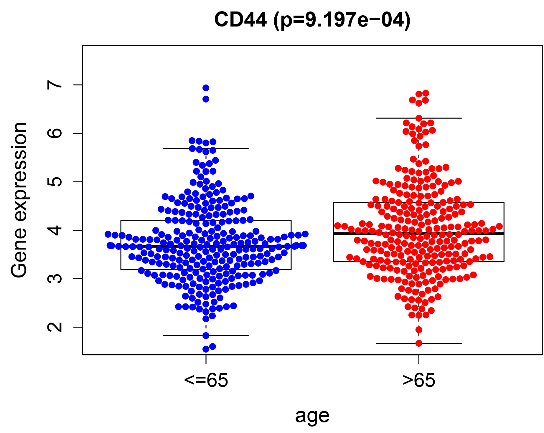

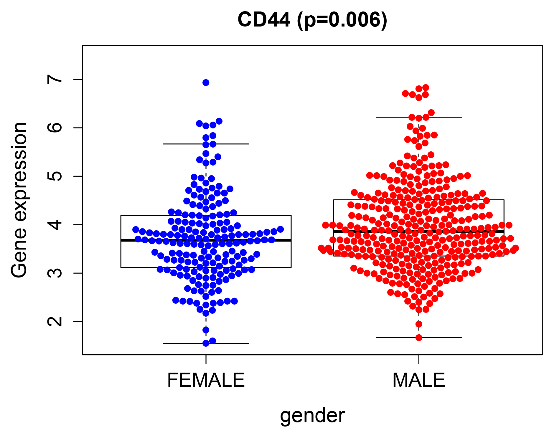


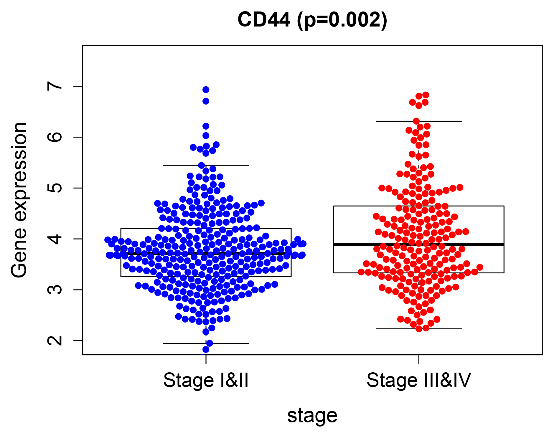

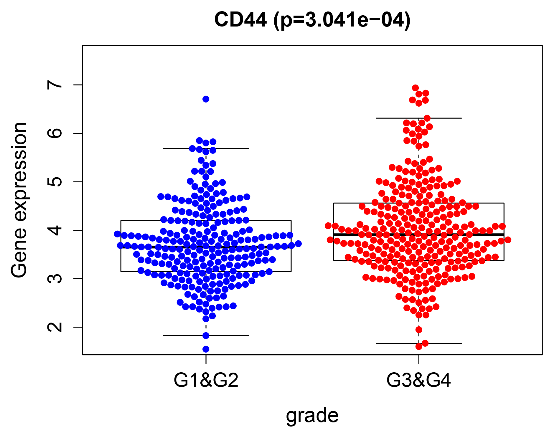


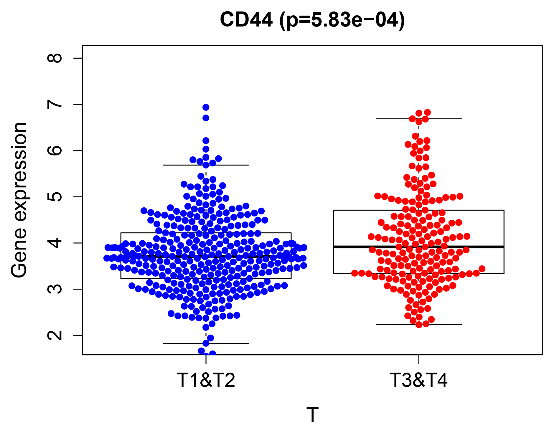

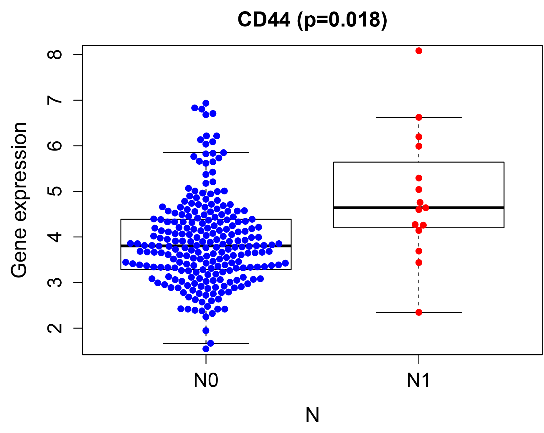


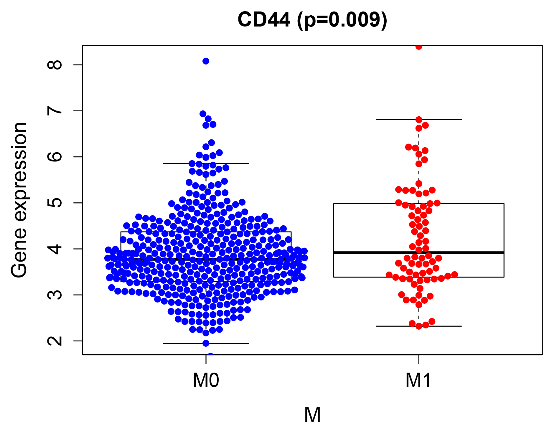


**Correlation between CD44 and clinicopathologic features**

**(age, gender, stage, grade, TNM-staging)**


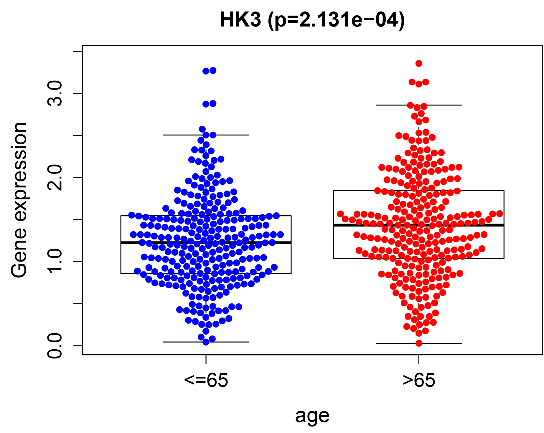

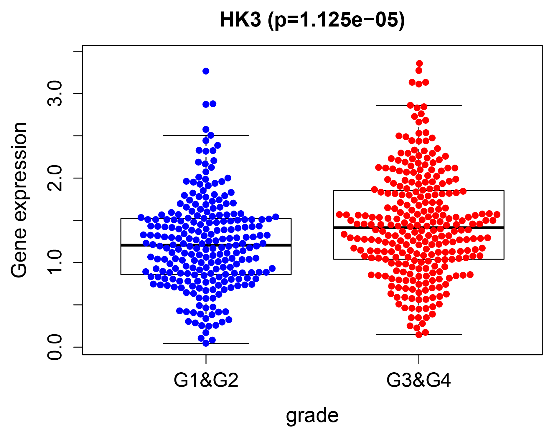


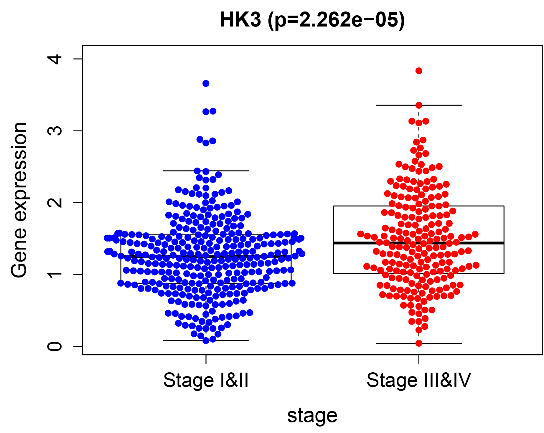

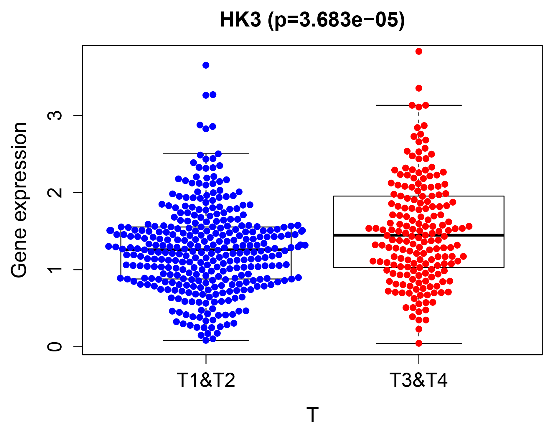


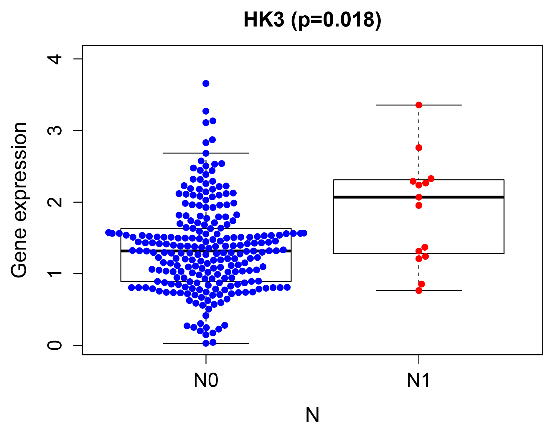

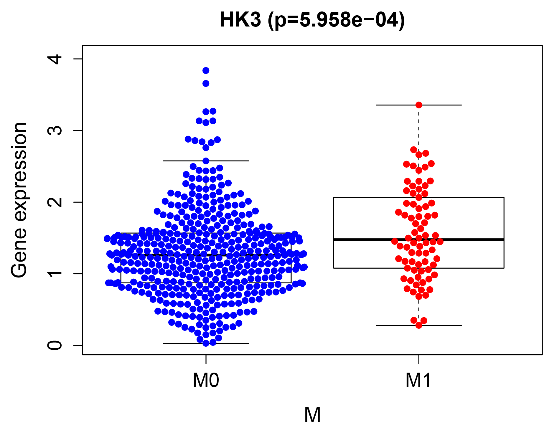


**Correlation between HK3 and clinicopathologic features**

**(age, gender, stage, TNM-staging)**

**
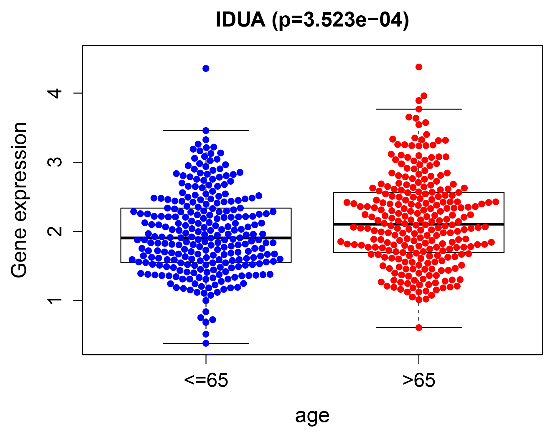
**
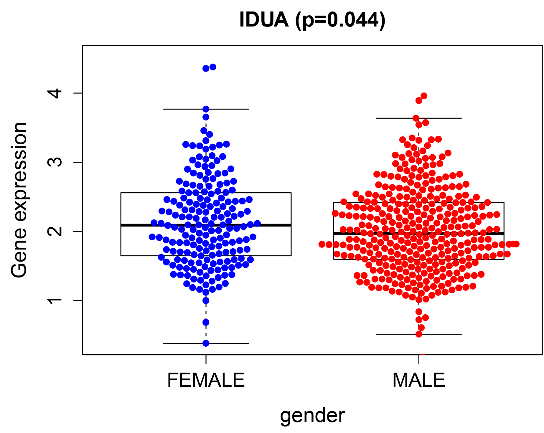


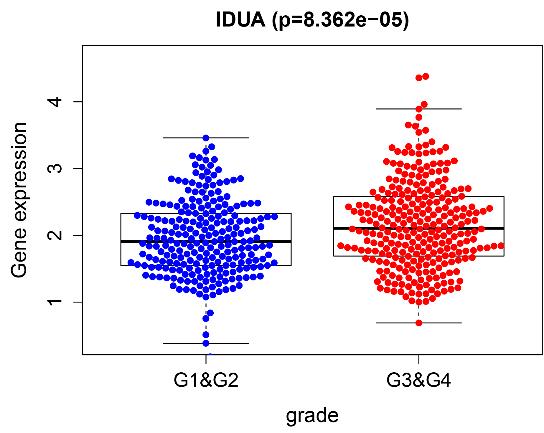

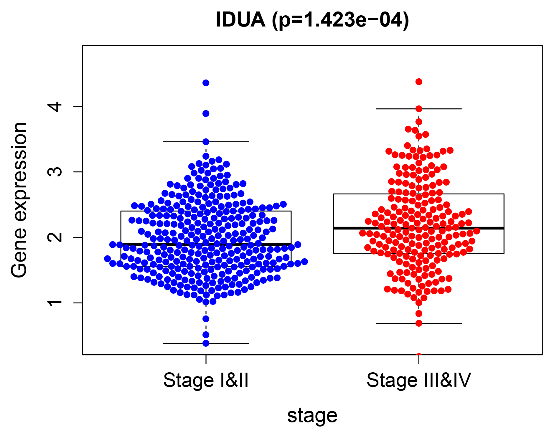


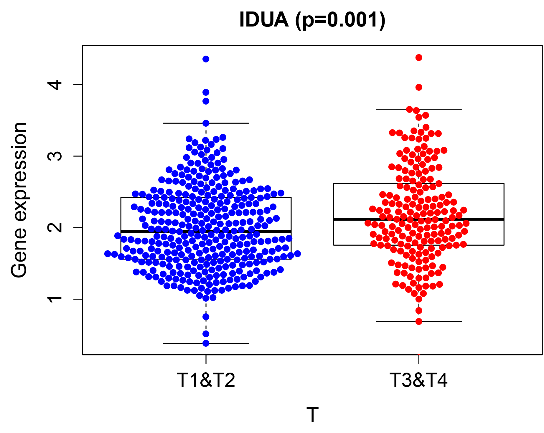

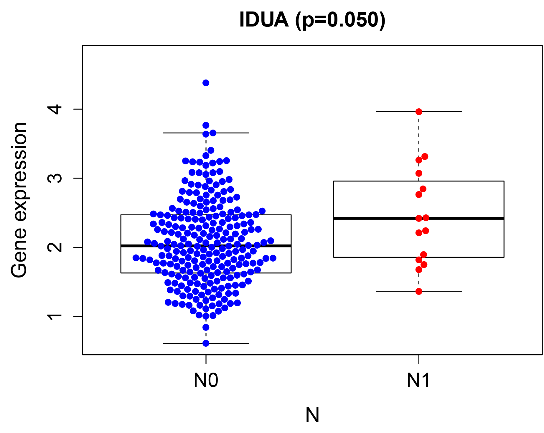


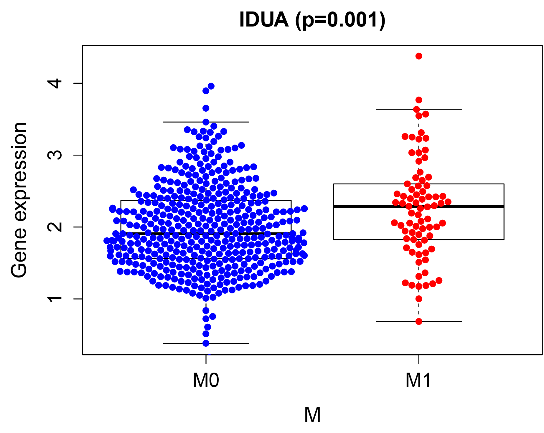


**Correlation between IDUA and clinicopathologic features**

**(age, gender, stage, grade, TNM-staging)**

**
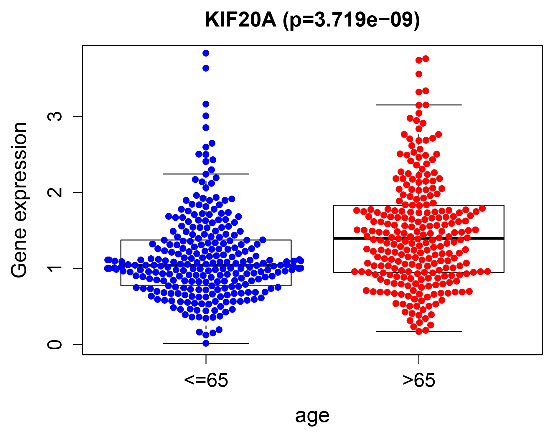
**
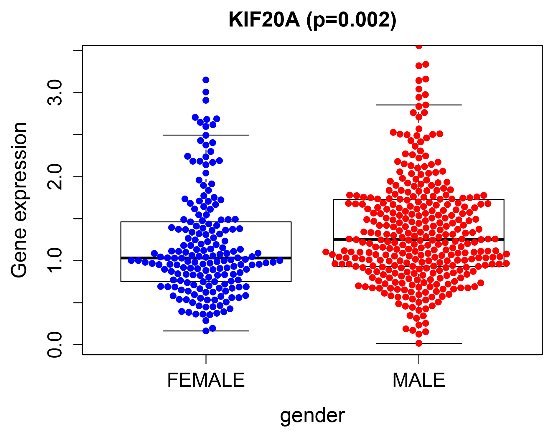


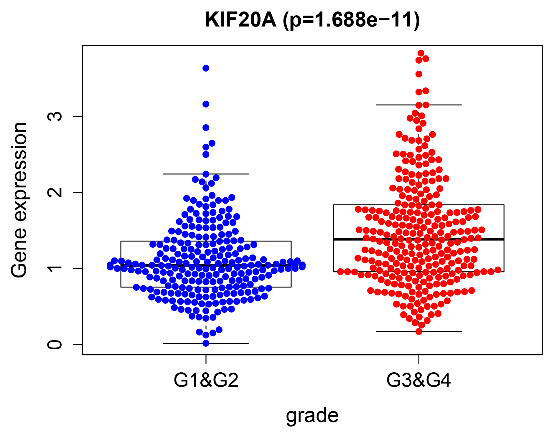

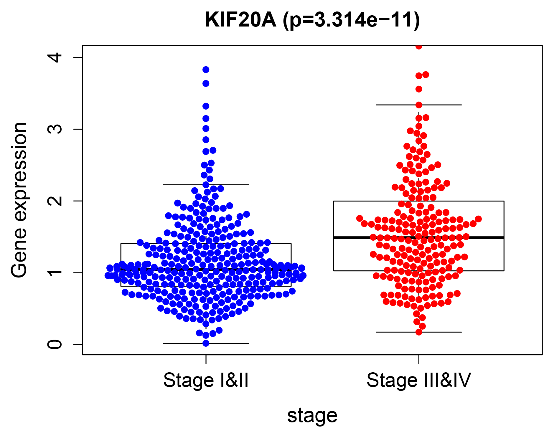


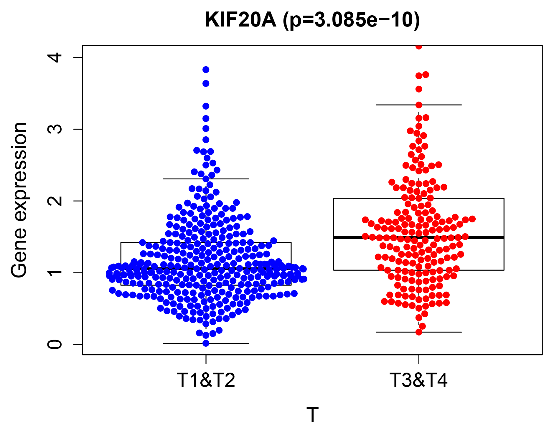

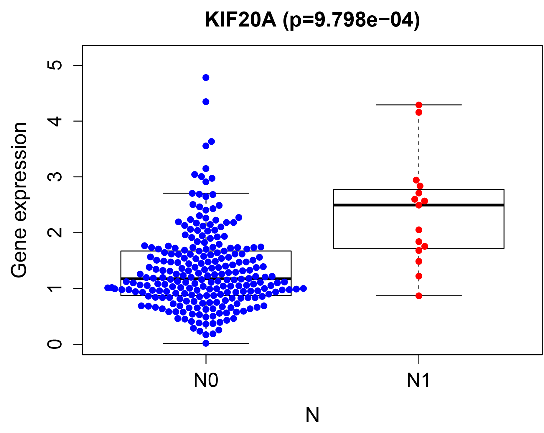


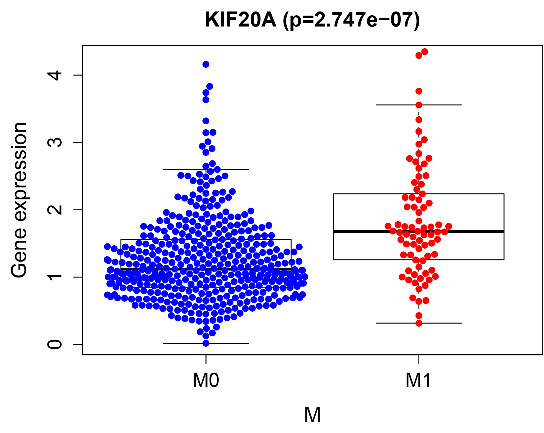


**Correlation between KIF20A and clinicopathologic features**

**(age, gender, stage, grade, TNM-staging)**


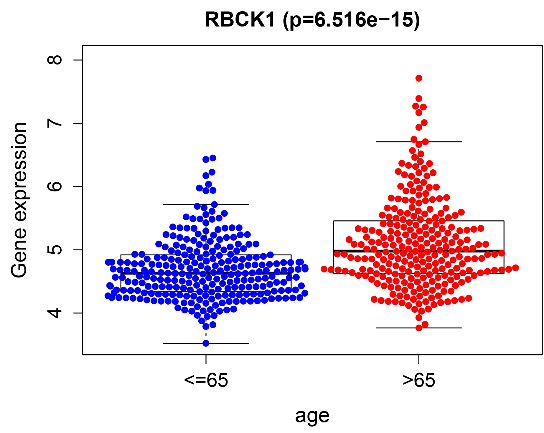

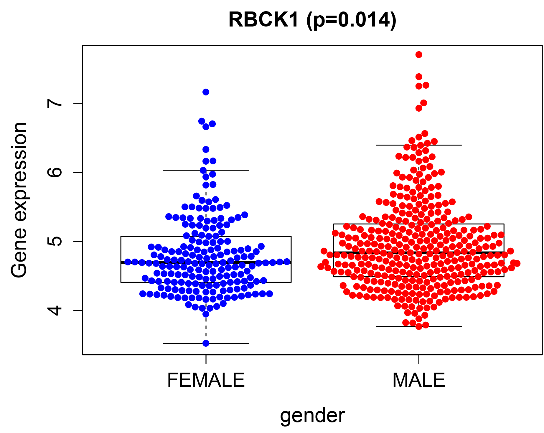


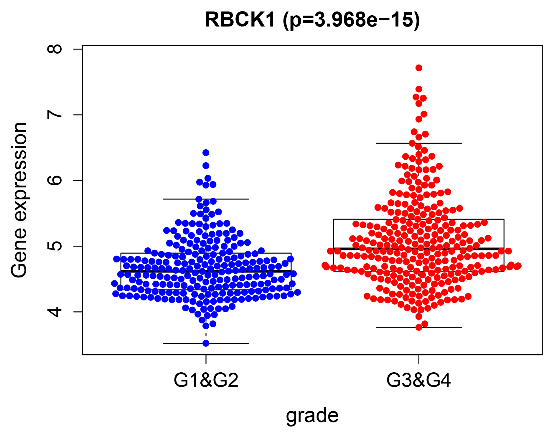

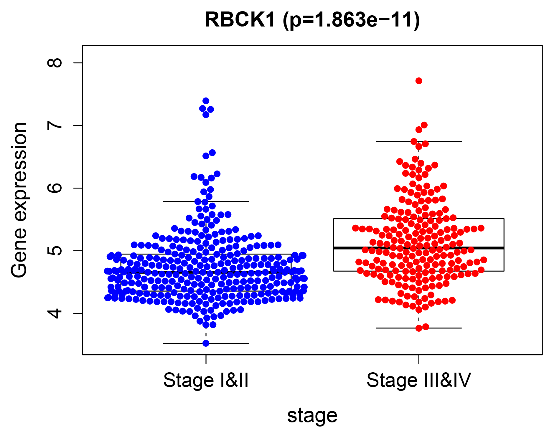


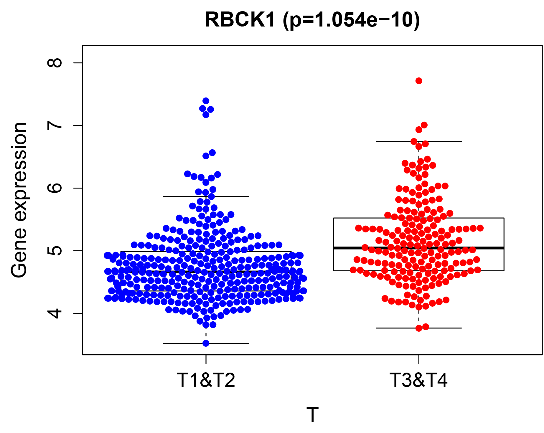

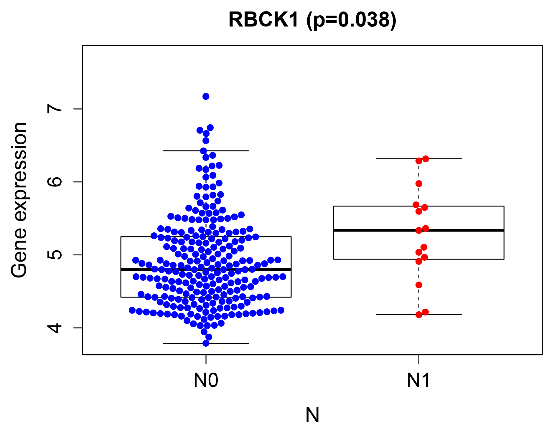


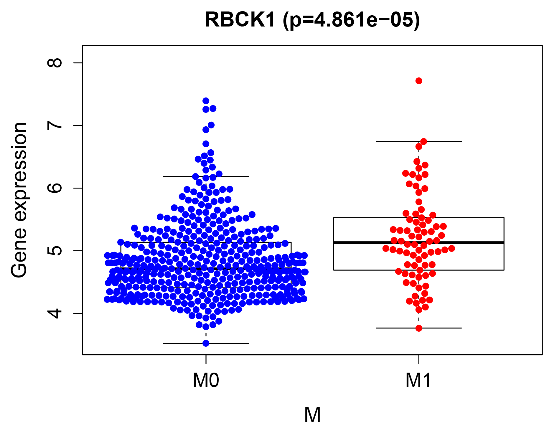


**Correlation between RBCK1 and clinicopathologic features**

**(age, gender, stage, grade, TNM-staging)**


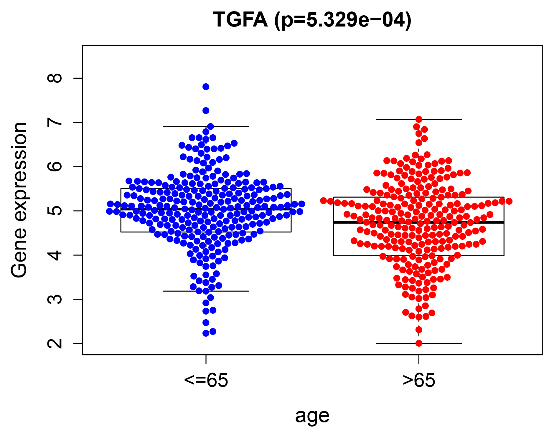

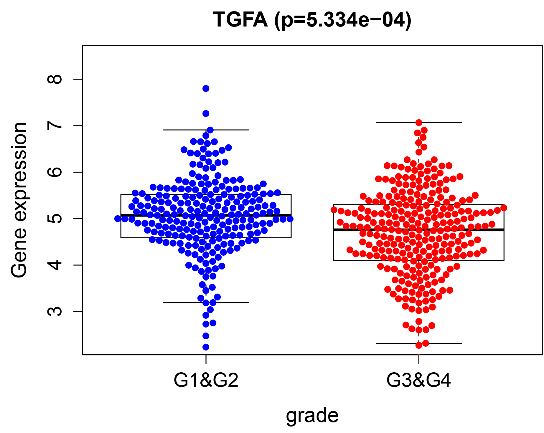


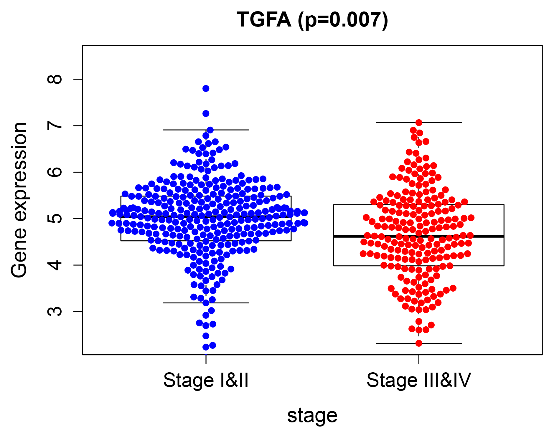

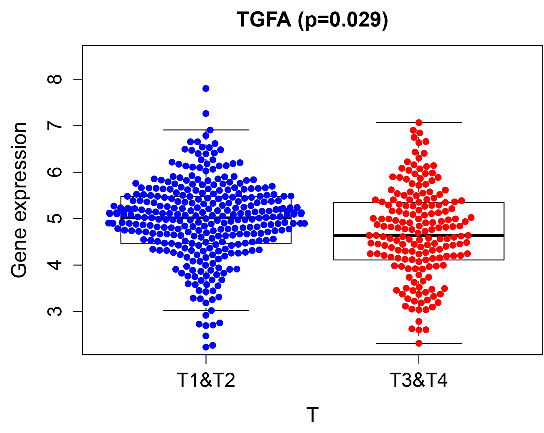


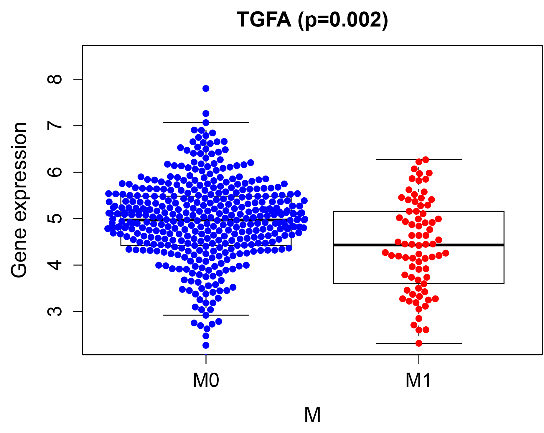


**Correlation between TGFA and clinicopathologic features**

**(age, gender, stage, grade, TM-staging)**


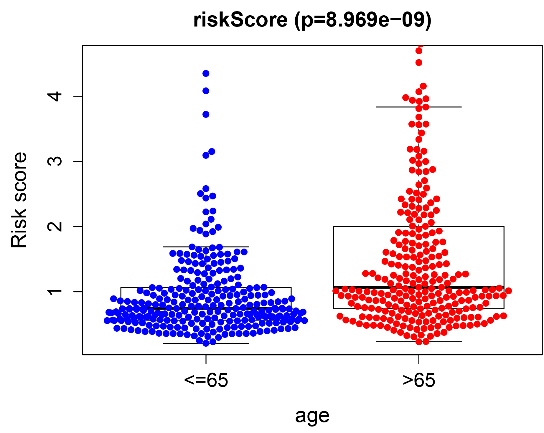

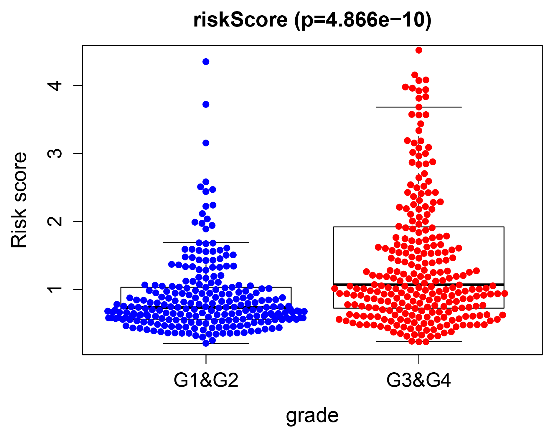


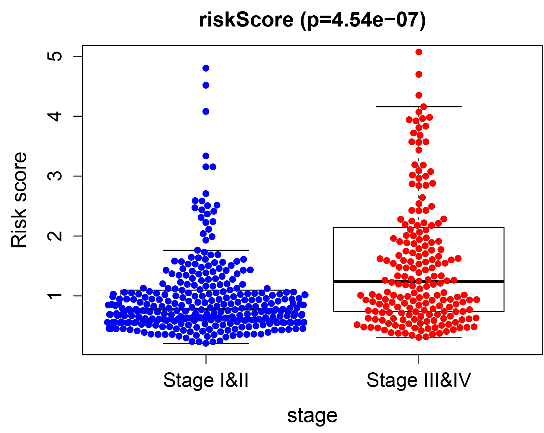

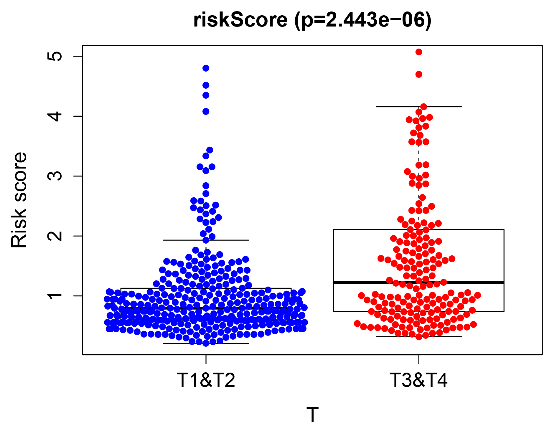


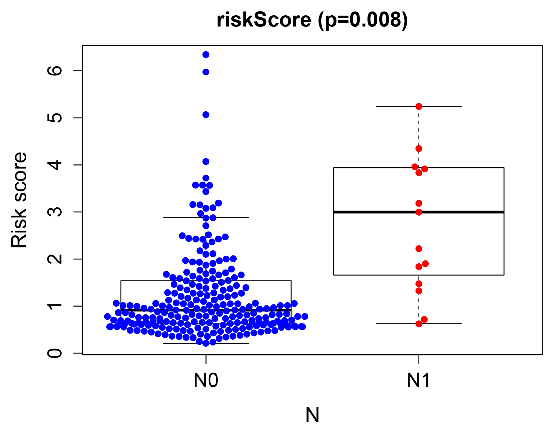

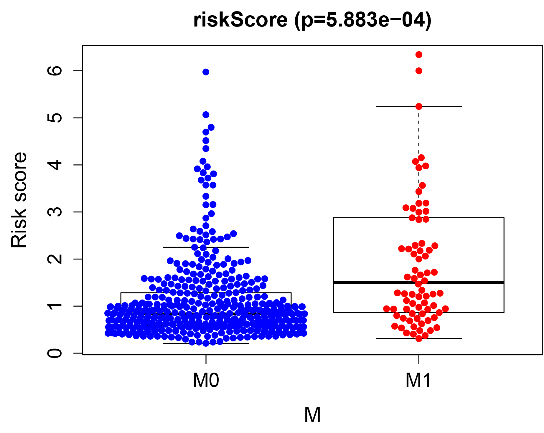


**Correlation between risk score and clinicopathologic features**

**(age, stage, grade, TNM-staging)**
